# Supplementary material for: Impact of financial literacy, mental budgeting and self control on financial wellbeing: Mediating impact of investment decision making
Source: PLoS One. 2023 Nov 14;18(11):e0294466. doi: 10.1371/journal.pone.0294466 (PMC10645357; doi:10.1371/journal.pone.0294466)
Supplement: S1 File — (DOCX) [file pone.0294466.s001.docx]

**Measurement Items**

**Financial Wellbeing**

| ***Serial*** | ***Items*** | ***SD*** | ***D*** | ***N*** | ***A*** | ***SA*** |
| --- | --- | --- | --- | --- | --- | --- |
| FWB1 | I get unsure by the lingo of financial experts. | 1 | 2 | 3 | 4 | 5 |
| FWB2 | I am anxious about financial and money affairs. | 1 | 2 | 3 | 4 | 5 |
| FWB3 | I tend to postpone financial decisions. | 1 | 2 | 3 | 4 | 5 |
| FWB4 | After making a decision, I am anxious whether I was right or wrong. | 1 | 2 | 3 | 4 | 5 |
| FWB5 | I feel secure in my current financial situation. | 1 | 2 | 3 | 4 | 5 |
| FWB6 | I feel confident about my financial future. | 1 | 2 | 3 | 4 | 5 |
| FWB7 | I feel confident about having enough money to support myself in retirement, no matter how long I live. | 1 | 2 | 3 | 4 | 5 |

**Financial Literacy**

| ***Serial*** | ***Items*** | ***SD*** | ***D*** | ***N*** | ***A*** | ***SA*** |
| --- | --- | --- | --- | --- | --- | --- |
| FL1 | I have better understanding of how to invest my money. | 1 | 2 | 3 | 4 | 5 |
| FL2 | I have better understanding of how to manage my credit use. | 1 | 2 | 3 | 4 | 5 |
| FL3 | I have a very clear idea of my financial needs during retirement. | 1 | 2 | 3 | 4 | 5 |
| FL4 | I have the ability to maintain financial records for my income and expenditure. | 1 | 2 | 3 | 4 | 5 |
| FL5 | I have little or no difficulty in managing my money. | 1 | 2 | 3 | 4 | 5 |
| FL6 | I have better understanding of financial instruments (e.g. bonds, stock, T-bill, future contract, option and etc.). | 1 | 2 | 3 | 4 | 5 |
| FL7 | I have the ability to prepare my own weekly (monthly) budget. | 1 | 2 | 3 | 4 | 5 |

**Mental Budgeting**

| ***Serial*** | ***Items*** | ***SD*** | ***D*** | ***N*** | ***A*** | ***SA*** |
| --- | --- | --- | --- | --- | --- | --- |
| MB1 | I have reserved money (budget) for different expenses, such as food, clothing, transportation, etc. | 1 | 2 | 3 | 4 | 5 |
| MB2 | I never spend more than a fixed amount on food, clothing, transportation, etc. | 1 | 2 | 3 | 4 | 5 |
| MB3 | If I spend more on one thing, I economize on other expenses. | 1 | 2 | 3 | 4 | 5 |
| MB4 | If I spend more than normal on one thing in 1 month, I spend less on other things in the next month. | 1 | 2 | 3 | 4 | 5 |

**Self-control**

| ***Serial*** | ***Items*** | ***SD*** | ***D*** | ***N*** | ***A*** | ***SA*** |
| --- | --- | --- | --- | --- | --- | --- |
| SC1 | I am good at resisting temptation. | 1 | 2 | 3 | 4 | 5 |
| SC2 | I am good at breaking my bad habits. | 1 | 2 | 3 | 4 | 5 |
| SC3 | I don’t do certain things that are bad for me even if they are fun. | 1 | 2 | 3 | 4 | 5 |
| SC4 | I have sufficient self-discipline. | 1 | 2 | 3 | 4 | 5 |
| SC5 | I act after thinking through all the alternatives. | 1 | 2 | 3 | 4 | 5 |
| SC6 | Regarding my future I want to leave as little as possible to fate. | 1 | 2 | 3 | 4 | 5 |
| SC7 | I often act in order to achieve something that may not result for many years. | 1 | 2 | 3 | 4 | 5 |
| SC8 | I believe it is important to save for the future. | 1 | 2 | 3 | 4 | 5 |
| SC9 | Regarding the future one always has to take into account that things may get worse. | 1 | 2 | 3 | 4 | 5 |

**Investment Decision Making Behavior**

| ***Serial*** | ***Items*** | ***SD*** | ***D*** | ***N*** | ***A*** | ***SA*** |
| --- | --- | --- | --- | --- | --- | --- |
| DMB1 | I do comparison when purchasing a product or service. | 1 | 2 | 3 | 4 | 5 |
| DMB2 | I pay all of my bills on time. | 1 | 2 | 3 | 4 | 5 |
| DMB3 | I keep a written or electronic record of my monthly expenses | 1 | 2 | 3 | 4 | 5 |
| DMB4 | I stayed within my budget or spending plan. | 1 | 2 | 3 | 4 | 5 |
| DMB5 | I paid off credit card balance in full each month. | 1 | 2 | 3 | 4 | 5 |
| DMB6 | I never crossed limits of my credit cards. | 1 | 2 | 3 | 4 | 5 |
| DMB7 | I made full payments of my loans. | 1 | 2 | 3 | 4 | 5 |
| DMB8 | I maintained an emergency savings fund. | 1 | 2 | 3 | 4 | 5 |
| DMB9 | I save money from every salary. | 1 | 2 | 3 | 4 | 5 |
| DMB10 | I save for a long term goal such as a car, education, home, etc. | 1 | 2 | 3 | 4 | 5 |
| DMB11 | I contribute money to a retirement account. | 1 | 2 | 3 | 4 | 5 |
| DMB12 | I bought bonds, stocks, or mutual funds. | 1 | 2 | 3 | 4 | 5 |

SD: Strongly Disagree

D: Disagree

N: Neutral

A: Agree

SA: Strongly Agree
