# Supplementary material for: Impact of financial literacy, mental budgeting and self control on financial wellbeing: Mediating impact of investment decision making
Source: PLoS One. 2023 Nov 14;18(11):e0294466. doi: 10.1371/journal.pone.0294466 (PMC10645357; doi:10.1371/journal.pone.0294466)
Supplement: Attachment — Submitted filename: PLOS ONE-D-23-18334_review SARGENT Financial Literacy Self Control.pdf.pdf [file pone.0294466.s002.pdf]

Impact of Financial Literacy, Mental Budgeting and Self Control on Financial Wellbeing:

Mediating Impact of Investment Decision Making

PONE-D-23-18334

Reviewer Comments

Overall: I think this is a major world problem and a significant research topic. I would like to see more than perception self-report measures to make the claims in this paper. Improving actual financial literacy vs. perceived financial literacy (self-report impressions that you have here) may lead to different recommendations. You lack important covariates. You may be able to rework this to be about perceptions influencing other perceptions and that might work. After all, you make the point that perceived FWB is more about the individual's interpretation, not actual financial status. You have a specific population (students) that may not have had extensive experience with financial decisions. That is a limitation (at least acknowledge it).

Introduction

You may be over playing the contribution to mental health. It is enough to indicate that financial stress is an important stressor. This can be shortened, perhaps substantially. You do not build up to your research questions (such that they are a logical next step in addressing financial well-being). For instance, you never mention mental budgeting or investment decisions prior to line 161 where they appear as a surprise. You have explained the problem but then you need to show how your work will address it. That link needs strengthening. You mention that many studies do not include a full array of variables, which is true, then you drop that aspect and have RQs that are lean (single variable), the very thing you criticize. You then have a paragraph about the importance of increasing FWB with factors not in your RQs (like retirement planning). Create a more concise organization: describe the problem (which is done extensively), how you address the problem (missing, RQs are not really doing the job here), the major findings and the implications given the findings. The specific RQs usually come after hypothesis.

Literature and Hypothesis

Just listing your variables and the literature for that variable is good but needs some introduction prior to jumping into them. Why start with FL? Is this for perceived and actual (measured) FL or just actual FL? In many models they have similar correlations on financial behaviors but mean slightly different things. Not sure what you mean here: "Managing finances for personal needs aligns with financial literacy from a practical perspective." (Line 193). Your comment: Financial literacy and financial well-being have been the subjects of separate research up until this point is unclear." (Line 198) Most of the literature on FL is impact on financial habits or financial outcomes, which you argue leads to FWB. Then you start discussing FWB, leaving FL. This seems like the place to put your RQ about FL.

In the second paragraph about mental budgeting you pivot to FL, (Line 230) and then back to mental budgeting. In line 235 you say mental budgeting has the "most" influence on financial behavior – more than any other variable – with one citation given. I do not agree as I look at Xiao & O'Neill (2018). The beta for that variable is much smaller than several other significant variables in the OLS on financial well-being. Further, other studies have found it to be

significant but not with a higher effect size over other variables such as education and income. The discussion on self-control does not mention how it was measured in these studies that found it correlated with positive financial outcomes. Is self-control nudgeable (vs. FL)?

Line 324: FL and self-control are significant in financial behavior and well-being. Seems to be leaving off some major variables (e.g., education, income, demographics). The discussion needs tighter organization. You then pivot to financial education and subjective FL in the mental budgeting section. Then parental education comes in (before individual's education?) and money attitudes.

I might introduce the full family of variables that link to financial behaviors and FWB and then one at a time discuss them, including how they are measured in the literature, leading to each RQ.

Method and Findings

Please put the results on Fig 1.

Wonder if students are typical of the national studies done in the literature? Most have not even started their careers or thought about emergency savings. I wonder about the ability to generalize from this.

Actual FL has been measured by the same 5 questions for decades and include, for example, the ability to figure compounding interest at 2% for one year. See Lusardi's line of research. I believe you have measure "perceived financial literacy," not actual FL. Perceived is the person's belief about financial competency. This differs from actual competency. For instance, for a discussion of the difference of these two, see:

Balasubramnian, Bhanu, and Carol Springer Sargent. "Impact of Inflated Perceptions of Financial Literacy on Financial Decision Making." Journal of Economic Psychology, vol. 80, 2020. https://doi.org/10.1016/j.joep.2020.102306.

You have failed to control for very important demographic information, especially education and income. Further, your FL variable, normally measured by an actual "test" of financial comprehension, is a FL perception self-report. Highly educated individuals may have better self-control and so you cannot tell which is giving you the result without controlling for these important covariates. You rightly criticize the literature for not including all the variables and then you do it too.

You may have a finding here but not the one you discuss. You have found that one's view about financial competence correlates with financial behaviors and financial well-being. And how do we know the direction? Maybe strong financial behaviors (paying things on line for instance) makes the individual feel more able (PFL) and in control (self-control). Could it be the perceptions follow from good habits and perceptions lead to FWB? You mention that two different individuals with the same financial circumstances can have different SWB perceptions. This would be a different paper but may be possible with this dataset.

Going forward, consider a more diverse sample, measuring AFL, and including covariates.

Small items to address:

- I would not characterize the cognitive aspects as a “personality” type. (line 155)
- Line 161: Remove “to”
