# Supplementary material for: Impact of financial literacy, mental budgeting and self control on financial wellbeing: Mediating impact of investment decision making
Source: PLoS One. 2023 Nov 14;18(11):e0294466. doi: 10.1371/journal.pone.0294466 (PMC10645357; doi:10.1371/journal.pone.0294466)
Supplement: Attachment — Submitted filename: Response to Reviewers.docx [file pone.0294466.s003.docx]

**RESPONSE TO EDITOR**

Contributions are rewritten clearly.

Relevant theory is added.

Implications of the findings are added.

**RESPONSE TO REVIEWER 1**

Concerns about objectives and subjective financial wellbeing are addressed and it is mentioned that author is interested in the later.

Introduction is shortened.

After discussing the problem, solution (i.e. role of selected factors) is also discussed.

Comments which were not matched as indicated by the reviewer are either removed or adjusted.

Education, income and demographics are ignored because of the sample, as respondents do not have very diverse characteristics.

**RESPONSE TO REVIEWER 2**

**Item 1: Why financial literacy and financial knowledge are not operationalized as multi-dimensional constructs?**

Operationalizing "financial literacy" as a uni-dimensional construct can have some advantages. Here are some reasons why author choose to operationalize financial literacy as a uni-dimensional construct:

Simplicity: A uni-dimensional approach simplifies measurement and analysis. It reduces the complexity associated with measuring multiple dimensions of financial literacy, making it easier to administer surveys, collect data, and analyze results.

Ease of Communication: Communicating and interpreting the results of a uni-dimensional financial literacy measure is straightforward. It allows for clearer communication of findings to policymakers, educators, and the general public.

Comparison: Uni-dimensional measures make it easier to compare individuals or groups based on a single scale, facilitating straightforward comparisons between different demographics, regions, or time periods.

Policy Implications: A uni-dimensional measure can be more effective for guiding policy decisions, as it provides a clear overall picture of financial literacy levels within a population. Policymakers may find it easier to target interventions when working with a single metric.

Apart from these reasons, the items chosen to measure financial literacy are broad and fulfill the researcher’s objective.

**Item 2: Financial literacy is measured by both subjective and objective approaches. Why authors adopted subjective approach only?**

Here's why author choose subjective approach:

Self-assessment and self-awareness: Subjective measures allow individuals to assess their own financial knowledge, skills, and confidence in managing their finances. This self-awareness can be valuable because it helps individuals recognize their own areas of weakness and take steps to improve their financial literacy. It can also serve as a motivation for individuals to seek out financial education and make positive changes in their financial behaviors.

Practicality and cost-effectiveness: Subjective measures are often easier and more cost-effective to implement than objective measures, which may require standardized tests or evaluations by financial experts. Subjective assessments can be administered through surveys or questionnaires, making them accessible to a wider range of people and organizations, including schools, employers, and financial institutions.

Cultural and contextual sensitivity: Financial literacy is not a one-size-fits-all concept. It can vary based on cultural, socioeconomic, and personal factors. Subjective assessments can capture the nuances of an individual's financial knowledge and attitudes, allowing for a more context-specific understanding of their financial literacy.

Focus on behavior and decision-making: Subjective measures often include questions about financial attitudes, behaviors, and decision-making, which are critical components of financial literacy. Understanding how individuals perceive and approach financial choices can provide valuable insights for designing targeted financial education programs and interventions.

**Item 3: Why Baron and Kenny's approach of mediation was adopted?**

Baron and Kenny's approach to mediation analysis is a widely used and influential method in the field of psychology and social sciences for investigating the mechanisms by which an independent variable affects a dependent variable through an intermediate variable (i.e., the mediator).

Clarity and Transparency: Baron and Kenny's approach provides a clear and step-by-step framework for conducting mediation analysis. It helps researchers systematically test the hypothesized mediation model, making the process more transparent and accessible.

Causal Inference: The approach emphasizes the establishment of causality in mediation relationships. It requires researchers to demonstrate that three conditions are met: (a) the independent variable significantly predicts the mediator, (b) the mediator significantly predicts the dependent variable while controlling for the independent variable, and (c) the direct effect of the independent variable on the dependent variable is reduced or becomes non-significant when the mediator is included in the model. This helps researchers make stronger claims about causality.

Practicality: Baron and Kenny's approach is relatively straightforward to implement. It does not require advanced statistical techniques or specialized software, making it accessible to a wide range of researchers.

Interpretability: The approach provides coefficients that are easily interpretable. Researchers can directly assess the size and significance of the indirect (mediation) effect, which is often of primary interest in mediation analysis.

In summary, Baron and Kenny's approach to mediation analysis is preferred for its simplicity, clarity, and emphasis on causality.

**RESPONSE TO REVIEWER 3**

Introduction is shortened.

The structure of the paper is given.

Limitations are identified.

Implications are discussed.
